# In ovo model with emu eggs as novel alternative to animal testing in preclinical imaging research

**DOI:** 10.1186/s13550-025-01314-7

**Published:** 2025-09-17

**Authors:** Olga Perkas, Marta Pomraenke, Veronika Porwoll, Christian Kühnel, Steffen Wiegand, Karl-Heinz Herrmann, Thomas Winkens, Martin Freesmeyer

**Affiliations:** 1 Clinic of Nuclear Medicine, Jena University Hospital, Am Klinikum 1, D-07747 Jena, Germany; 2 Jena University Hospital, Institute of Diagnostic and Interventional Radiology, Medical Physics Group, Philosophenweg 3, D-07743 Jena, Germany

**Keywords:** In ovo model, PET/CT, Emu egg, Preclinical imaging, 3R, Animal testing

## Abstract

**Background:**

Large-size in ovo models are receiving increasing attention since they comply with the 3R requirements (Replacement, Reduction and Refinement) by limiting the number of fully developed laboratory animals. In preclinical imaging research, a specific advantage is that they do not require dedicated scanners for small animals (expensive and rarely available) but are suitable for imaging studies by scanners used for clinical examinations. The present study evaluated large-sized fertilized emu eggs as a candidate model for preclinical imaging research in nuclear medicine by [^18^F]FDG-PET/CT, aiming to increase the repertoire of alternative models to conventional animal testing.

**Results:**

Of 31 fertilized eggs, 18 eggs had viable peripheral vasculature available for vessel detection via MRI or CT. Both modalities provided reliable information on location and dimension of target blood vessels. Optimization of catheterization proved challenging, and only 5 [^18^F]FDG-PET/CT scans were entirely successful in demonstrating the expected biodistribution pattern. In vivo and ex vivo organ activity showed a statistically significant correlation (Spearman’s Rho: 0.9091; *p* = 0.00004).

**Conclusion:**

The emu egg model is suitable for preclinical imaging research with clinical scanners. Considering the shorter seasonal availability but longer incubation period of fertilized emu eggs, this model is a valid complement to the recently introduced ostrich egg model, available only in warm periods. In combination, these models offer a year-round flexibility for in ovo imaging research.

**Supplementary Information:**

The online version contains supplementary material available at 10.1186/s13550-025-01314-7.

## Background

Large-size in ovo models are receiving increasing attention since they comply with the 3R requirements (Replacement, Reduction and Refinement) by limiting the number of fully developed laboratory animals [1, 2]. In preclinical imaging research, a specific advantage is that they do not require dedicated scanners for small animals (expensive and rarely available) but are suitable for imaging studies by scanners used for clinical examinations. Embryonated ostrich eggs were recently introduced as a suitable alternative to established mammal models and relevant data have already been obtained with cancer xenograft models [3, 4]. One limitation of the ostrich egg model, however, is that the eggs are available only in the warm season (March to September in the northern hemisphere), hence year-round research is not possible. In contrast, eggs from emu (Dromaius novaehollandiae) are available from autumn to spring [5], and are well established in research fields such as nutrition [6–8], embryonal development [9, 10], archeology [11], and physical characteristics, e.g., fracture resistance [12].

**Fig. 1:**
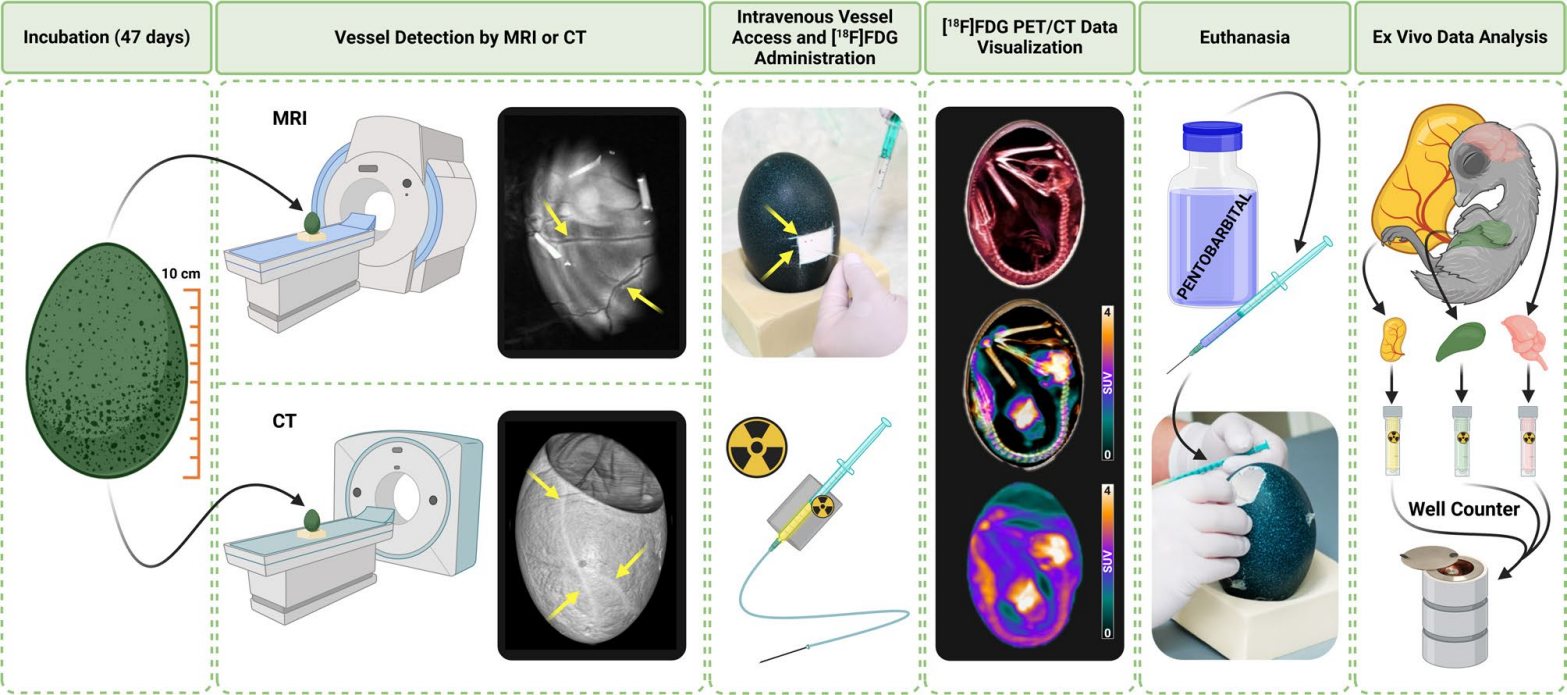
Flowchart illustrating entire workflow of emu egg model (Created in https://BioRender.com)

The present study evaluated large-sized fertilized emu eggs as a candidate model for preclinical imaging research in nuclear medicine. [^18^F]FDG was selected for this purpose as a widely available, commonly used, inexpensive, but key radiopharmaceutical used in clinical examinations for the study of energy metabolism in vivo (Fig. 1).

## Methods

**Fig. 2:**
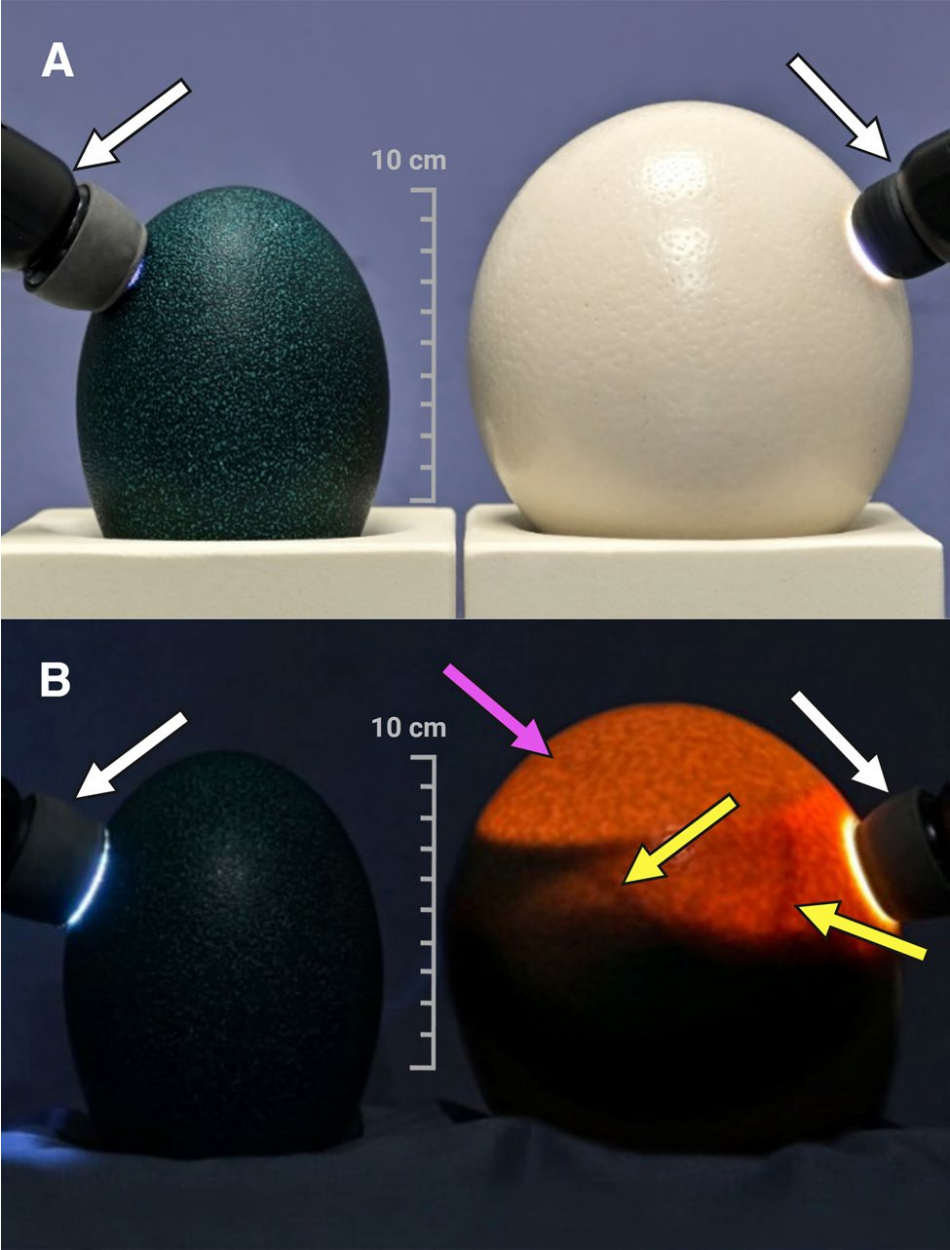
Diaphanoscopy settings: emu (left side) and ostrich (right side) eggs with candling lamps (white arrows). (**A**) In daylight no egg content is visible. (**B**) In the dark no content is visible in the emu egg, compared to visibility of air cell (pink arrow), blood vessels (yellow arrows), and embryonal structures in the ostrich egg (Created in https://BioRender.com)

Compared to ostrich eggs, challenging aspects of emu eggs include a shorter laying season, scarcity of husbandry farms [13], and especially a dark green opaque eggshell that hinders simple diaphanoscopy for vessel identification (Fig. 2). Thus, this study paid special attention to overcome these specific limitations, while maintaining comparability with the ostrich model, as complementary applications over the different laying seasons are expected to expand the possibility of year-round in ovo imaging research [3, 4, 14].

### Emu eggs, incubation conditions and physiologic monitoring

Emu eggs were purchased from a specialized farm (Emu-Hügel, Bad Schmiedeberg, Germany) during a single laying season from December until March. The number of emu eggs obtained from the farm was determined by reproduction activity and thus, each week between 2 and 6 eggs were available for artificial incubation. Detailed information on incubation conditions is provided in the Supplementary material 1. The first day of incubation was defined as developmental day zero (DD 0). On DD 25 fertilization was assessed by CT scan and viability by carbon dioxide (CO_2_) output (the latter repeated also before any further MRI and CT scanning according to Neuschulz [15]). Non-fertilized eggs and dead-in-shell embryos were immediately discarded. The remaining embryos were sacrificed at the latest on DD 47 (immediately after PET/CT imaging) using lethal doses of sodium pentobarbital (500 mg per embryo; Euthadorm 500 mg/ml; CP-Pharma Handelsgesellschaft mbH, Burgdorf, Germany) [16]. During imaging sessions and transfer between study sites, the eggs were kept warm using commercial cherry stone pillows. Rigid upright positioning during preparation, intravenous tracer administration, and imaging was kept by placing the eggs on polyurethane bases (NECURON 100, NECUMER GmbH, Bohmte, Germany) adjusted with a central pit.

### Vessel visualization

**Fig. 3:**
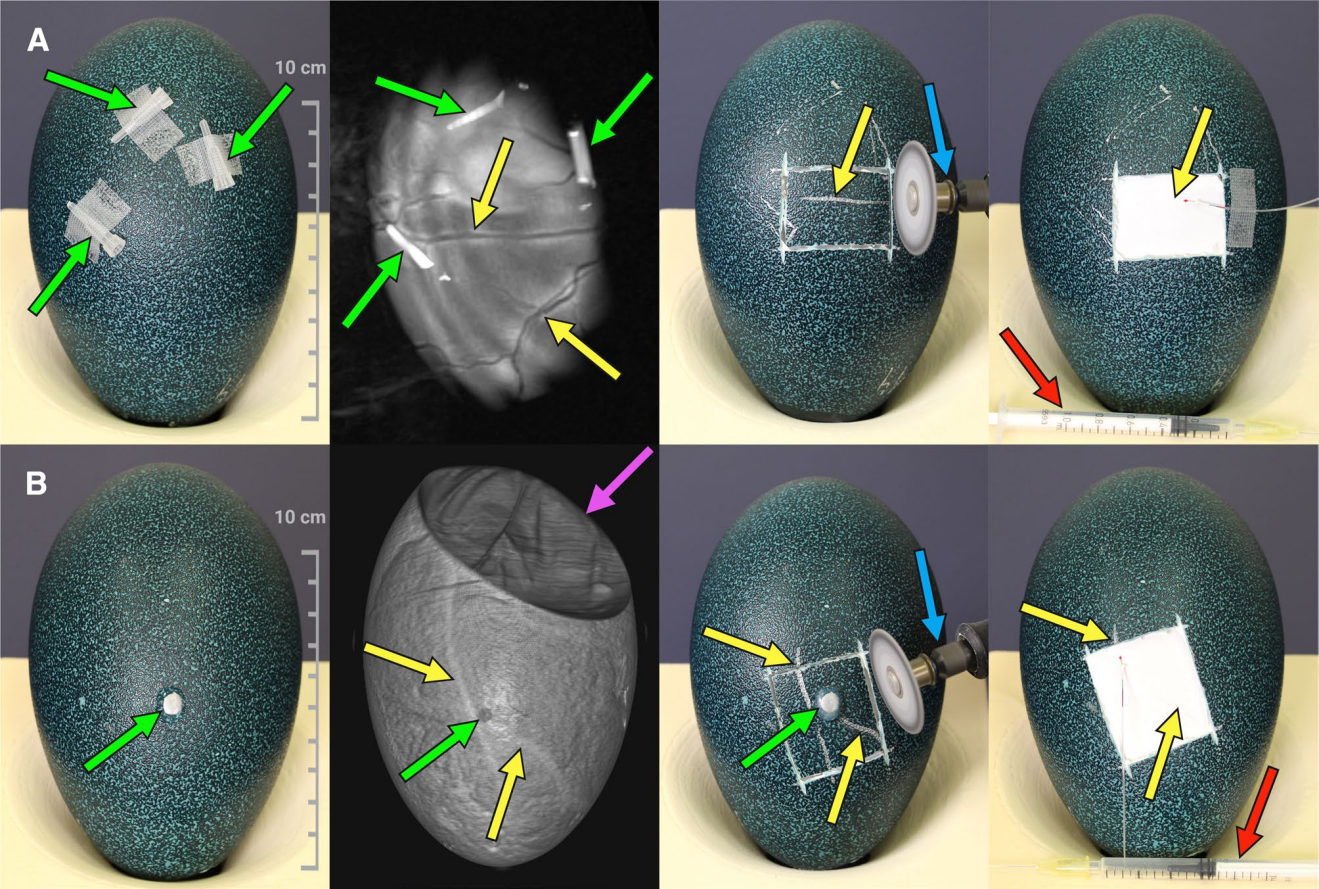
Preparation of emu eggs on DD 47 for intravenous administration of [^18^F]FDG. (**A**) Workflow using MRI and gadolinium-doped markers (green arrows). MRI scan enabled detection of blood vessels (yellow arrows). (**B**) Workflow using CT and glue markings (green arrows). Volume-rendered technique (VRT) was used for vessel detection (yellow arrows)

Intravenous administration of [^18^F]FDG required careful localization of target vessels of the chorioallantoic membrane (CAM). Due to the dark-green eggshell, common diaphanoscopy was not feasible (as detailed in Fig. 2) [4, 17], hence CT and MR imaging were used in alternative. MRI was performed using a 1.5 Tesla MR scanner (Magnetom Avanto, Siemens Healthineers, Erlangen, Germany) equipped with a 4-channel breast coil. A two-dimensional T2-weighted turbo spin echo (TSE) sequence was employed to acquire the images. The imaging parameters were as follows: in-plane resolution of 0.5 mm x 0.5 mm, slice thickness of 2 mm, and a total of 15 slices covering the region of interest. The field of view (FoV) was set to 224 mm, with a repetition time TR = 4550 ms and an echo time TE = 47 ms. The phase resolution was adjusted to 80%, and the total acquisition time (TA) for the sequence on one tangential plane was 74 s. A preliminary axial scan covering the entire egg was performed to obtain an anatomical overview. Then, four tangential imaging blocks were acquired at the outer periphery of the egg, positioned on four sides to achieve enhanced localization of peripheral vasculature. The most promising vessel was then identified, and small markers containing gadolinium-doped water were placed on the eggshell above the chosen vessel (as detailed in Fig.  3A). The markers were iteratively adjusted using successive MRI scans to refine their placement, and served as orientation to mark the eggshell area to be fenestrated for catheterization. MRI was performed on DD 42–46, depending on scanner availability and personnel resources.

In both cases successful eggshell fenestration was performed with a rotating cutter (blue arrow), allowing subsequent catheterization with a self-made injection system prefilled with saline (Created in https://BioRender.com).

CT scans were performed on DD 46–47 on a clinical scanner (Biograph mCT40, Siemens Healthineers, Erlangen, Germany). Data acquisition was performed with following parameters: tube voltage 120 kV; tube current-time product 200 mAs; increment 0.3 mm; slice thickness 0.6 mm; pitch 0.6; reconstruction kernel H20; scan duration 34 s. Images were reconstructed using filtered back projection and a reconstruction matrix of 512 × 512 into slices of 0.6 mm thickness. Prior to CT imaging, three hot-melt glue markings were applied to the eggshell for visual orientation (as detailed in Fig. 3B). Based on the acquired images, candidate vessels were marked on the eggshell. The CT parameters were kept identical for both vessel visualization and anatomical co-registration as part of PET/CT scans.

### Vessel access

After determining the eggshell area to be fenestrated, a rectangle of approximately 3 × 4 cm was outlined on the eggshell using a common paint marker. Careful cutting with a rotating cutter (Dremel, Bosch Powertools B.V., Breda, Netherlands) allowed removal of the marked rectangular piece of eggshell without damaging the shell membrane (Fig. 3). Then, a self-made peripheral venous catheter assembled from two 30gauge sterile hypodermic needles (Sterican, B. Braun SE, Melsungen, Germany) connected end-to-begin by a polyethylene tube with an inner diameter of 0.28 mm (BD Intramedic, Fisher Scientific GmbH, Schwerte, Germany) was inserted in the target vesselin. Correct intraluminal placement was confirmed by aspirating blood into the saline-filled injection system and flushing with saline. The polyethylene tube was fixed on the eggshell (Fig. 3A) to prevent needle dislocation during PET/CT scanning.

### Radiopharmaceutical

10 MBq of [^18^F]FDG (Alliance Medical f-con GmbH, Holzhausen an der Haide, Germany), maximum volume 0.5 ml in 1-ml syringes (Omnifix-F, B.Braun SE, Melsungen, Germany) were administered. Before and after injection, the activity was determined using a standard dose calibrator (ISOMED 2010, NUVIA Instruments GmbH, Dresden, Germany) and corrected for background activity.

### PET/CT i`maging

PET data acquisition was started simultaneously with [^18^F]FDG administration as dynamic list-mode over 60 min (two data sets, each 30 min long). Iterative technique (4 iterations; 12 subsets; image size 512 × 512; zoom factor 2; Gaussian filter) and reconstruction method TrueX (Siemens Healthineers) were utilized for data reconstruction. List-mode data sets were reconstructed in different time frames, i.e., the first data set (minute 0–30) in 5-min timeframes and the second set (minute 31–60) in 10-min time frames. All images were investigated with the software syngo.via (VB60A, Siemens Healthineers) used also in clinical routine. Small spheric volumes of interest (VOIs; 0.3 ml) were placed in liver, brain, yolk, and cloaca. The VOI activity was expressed as kBq/ml and SUV and analyzed over time to determine the in vivo distribution of [^18^F]FDG.

### Ex-vivo organ activity

After sacrificing the embryos, liver, brain, and yolk were removed and weighed. Radioactivity levels were measured using a well counter (ISOMED 2100, NUVIA Instruments GmbH, Dresden, Germany). The activities were then calculated and decay-corrected to analyze the correlation between in vivo and ex vivo levels.

### Statistics

Data were analyzed using Excel (Microsoft Excel 2016, Microsoft Corporation, Redmont, WA, USA). Correlations between in vivo and ex vivo data were calculated using the Spearman’s correlation coefficient test. Bland-Altman-plots provided visualization of comparisons, outliers, and bias. P values < 0.05 were considered significant for all comparisons.

## Results

**Fig. 4:**
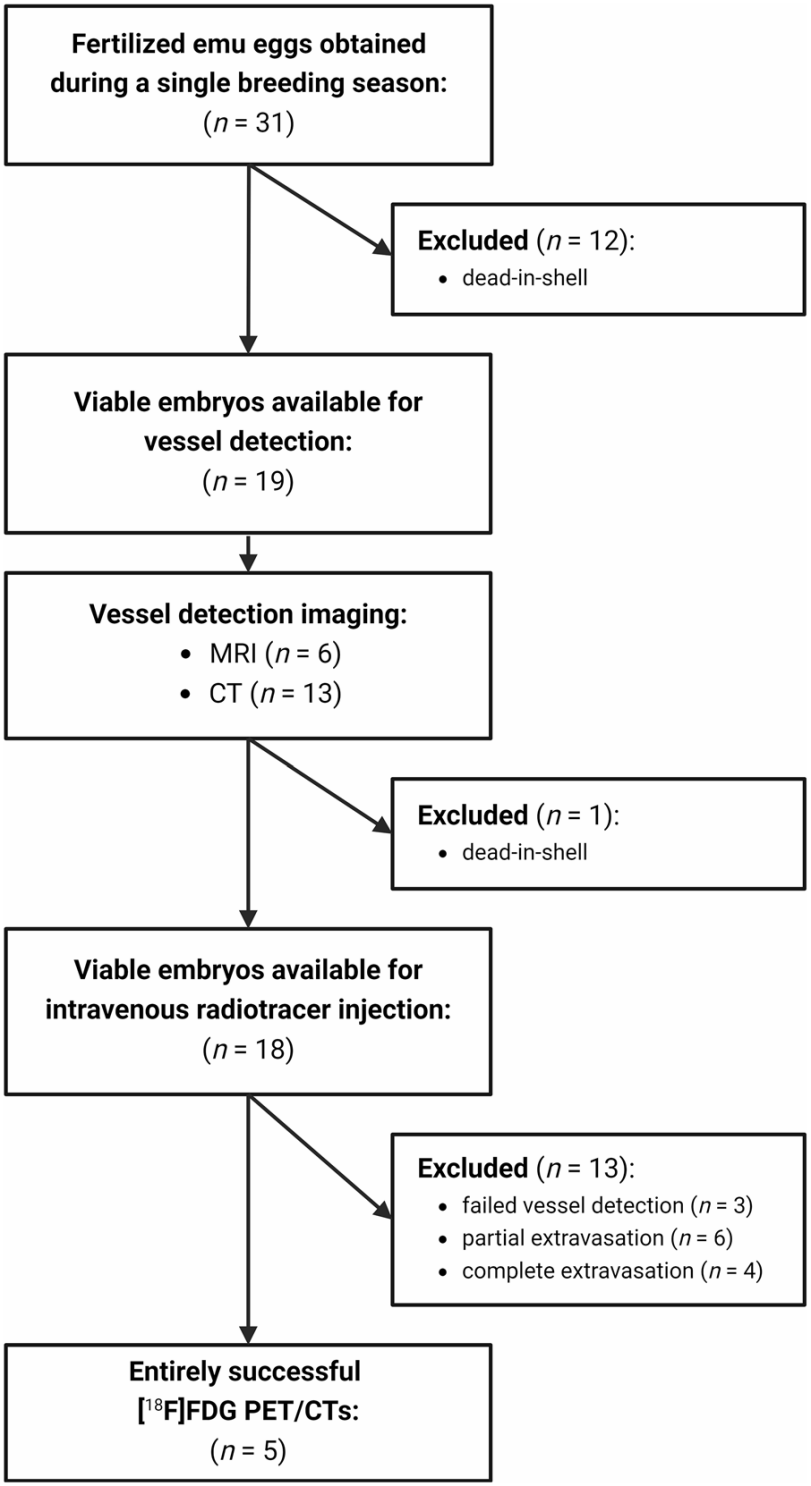
Flowchart illustrating entire inclusion process of emu eggs (Created in https://BioRender.com)

A total of 31 fertilized emu eggs (598.7 ± 5.6 g; range 424–683 g) were obtained during a single breeding season. Fertilization was confirmed by embryo visualization via CT scan on DD 25. In the last week of breeding (DD 41 until DD 46), 19 of 31 eggs (61.3%) showed viability by adequate CO_2_ output and were therefore available for vessel detection by MRI or CT. After exclusion of one late deathinshell embryo on DD 42, 18 emu eggs (58.1%) were available for PET/CT scanning on DD 47. Overall, 13 of 31 fertilized eggs (42%) died in shell (Fig. 4). Uncomplicated PET/CT imaging was successful in 5 cases (13.5%).

### Vessel visualization for catheterization

**Fig. 5:**
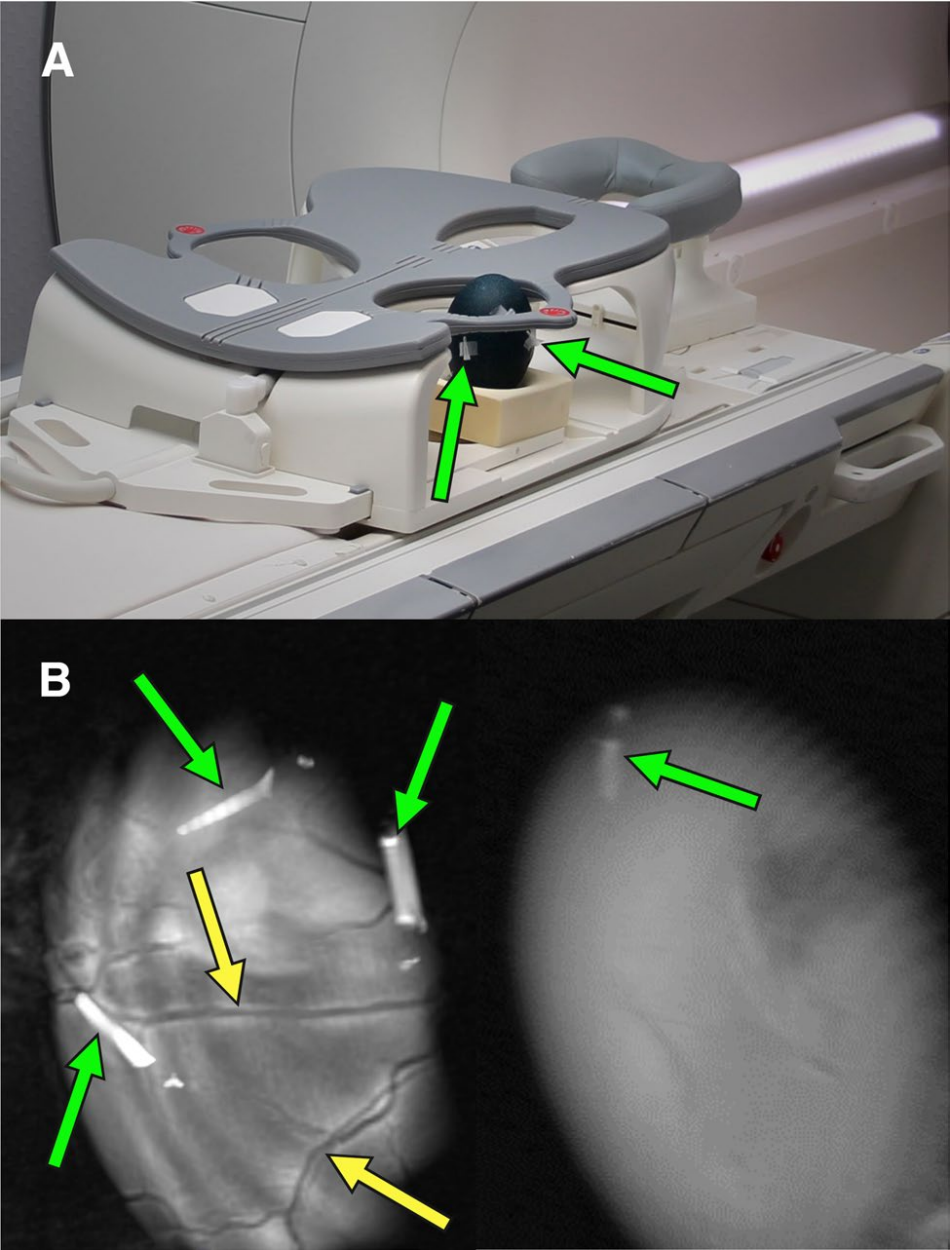
(**A**) Positioning of an emu egg in a breast coil for MRI. Gadolinium-doped markers (green arrows) were placed on the eggshell. (**B**) The MRI scans with gadolinium-doped markers (green arrows), show a viable embryo with visible blood vessels (left side; yellow arrows) and a dead-in-shell egg (right side) devoid of vessels (Created in https://BioRender.com)

**Fig. 6:**
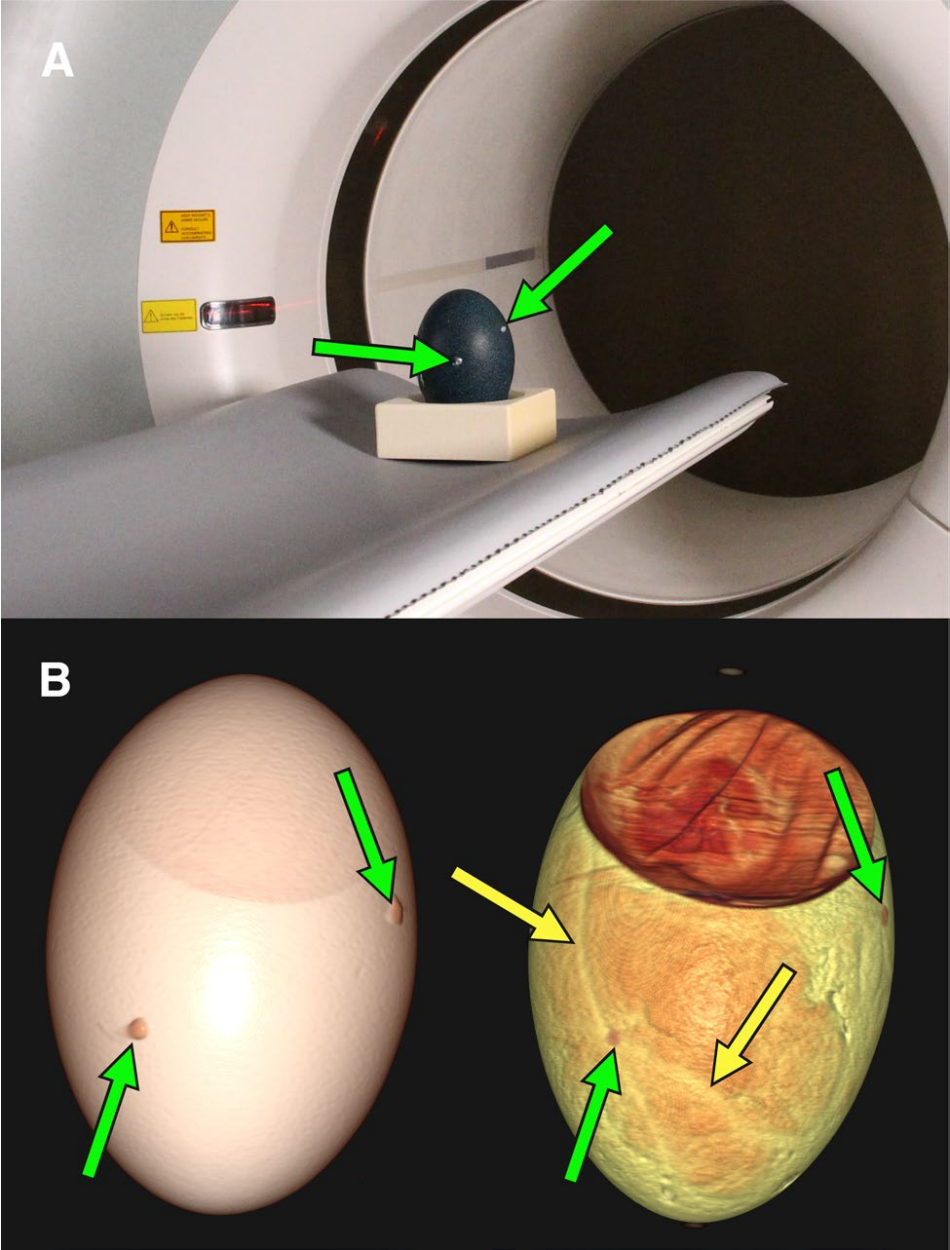
(**A**) Positioning of an emu egg for CT scan. (**B**) Three-dimensional reconstruction in VRT shows the eggshell (left side) with CT markers (green arrows) and blood vessels (yellow arrows) beneath the eggshell (right side) (Created in https://BioRender.com)

MRI (Fig. 5) and CT (Fig. 6) allowed a similarly good visualization of vessels beneath the darkgreen eggshell. However, while the acquisition time by CT was only 34 s, the MRI scan took at least 5 min per egg. Considering the entire time from and back to the incubator, the MRI took a minimum of 2 h (range 2–5 h), i.e., at least four times as long compared to the approximately 30 min required for transport, preparation, positioning, and data acquisition by CT, although this difference was affected by the local distance between the CT and MRI facilities.

### Vessel access

While removal of the targeted eggshell area succeeded in all 18 viable cases, in 3 cases no vessel was found in the marked area (3/18; 16.7%). Of the remaining 15 eggs, an entirely successful PET/CT imaging was achieved only in 5 cases (5/18; 27.8%). In the other 10 cases, an initial vessel access was achieved, but damage to the vessel wall or catheter slipping out of the lumen resulted in complete (6/18; 33.3%) or partial (4/18; 22.2%) extravasation of the radiotracer, as evidenced by PET/CT (see Fig. 4 for details).

### PET/CT imaging and organ activity

**Fig. 7:**
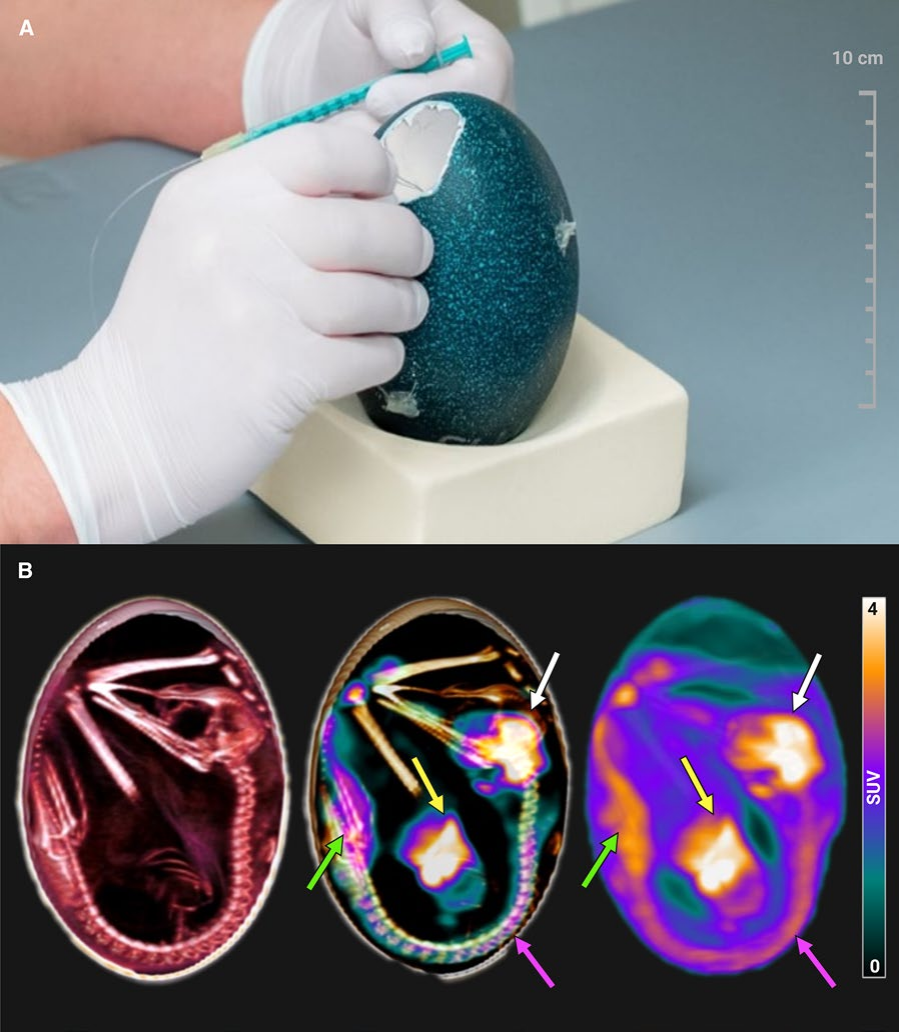
(**A**) Preparation for intravenous administration of [^18^F]FDG. (**B**) Representative PET/CT with typical anatomical (left side), metabolic (right side), and fusion images (middle). [^18^F]FDG uptake was evident in brain (white arrows), liver (yellow arrows), kidneys (green arrows), and spinal cord (pink arrows) (Created in https://BioRender.com). Additional visualizations are shown in Supplementary material 4, 5, and 6

A representative PET/CT image is shown in Fig. 7. In reconstructed PET/CT data, the [^18^F]FDG biodistribution over time proved similar to that observed in the ostrich model [4], i.e., the liver showed an initial peak activity followed by a continuous decrease, whereas the brain uptake increased in the first 15 min and then started to slowly decrease. The cloaca showed a steady activity increase over time, reflecting the expected urinary excretion. Inside the yolk mass, the activity was nearly 10-fold lower than any minimum organ concentration, and remained nearly unchanged through the acquisition time.

The results of decaycorrected ex vivo organ activity (Table 1) confirmed the in vivo measurements and showed a highly significant correlation (Spearman’s Rho: 0.9091; *p* = 0.00004; mean difference ratio: 0.92; lower limit of agreement: 0.65; upper limit of agreement: 1.20).

**Table 1:** Comparison of in vivo and ex vivo organ activity

Organ activity concentration [kBq/ml] measured in vivo by PET over 8 time intervals after administration											Organ acitivity concentration [kBq/g] measured ex vivo by well counter, decay corrected
**Time Interval**	[minutes]	1–5	6–10	11–15	16–20	21–25	26–30	31–40	41–50	51–60	70–80
**(Egg (whole)**	*Mean*	*11.20*	*11.76*	*11.94*	*12.05*	*12.13*	*12.24*	*12.38*	*12.48*	*12.55*	*NA*
	*SD*	*3.13*	*3.18*	*3.15*	*3.16*	*3.17*	*3.20*	*3.25*	*3.27*	*3.30*	*NA*
**Liver**	*Mean*	*67.07*	*55.13*	*49.79*	*46.08*	*44.55*	*44.54*	*42.18*	*41.63*	*40.83*	*35.96*
	*SD*	*9.87*	*15.99*	*15.54*	*14.77*	*15.11*	*14.98*	*14.51*	*14.76*	*14.41*	*12.07*
**Brain**	*Mean*	*38.78*	*49.06*	*50.53*	*50.36*	*49.57*	*49.13*	*49.24*	*49.35*	*48.82*	*56.35*
	*SD*	*12.51*	*14.32*	*14.38*	*13.24*	*13.84*	*13.58*	*13.60*	*14.07*	*13.72*	*14.89*
**Cloaca**	*Mean*	*15.55*	*24.15*	*26.95*	*27.65*	*36.71*	*41.44*	*41.34*	*47.38*	*51.95*	*NA*
	*SD*	*5.02*	*7.11*	*6.29*	*5.97*	*12.57*	*18.90*	*24.81*	*27.20*	*31.43*	*NA*
**Yolk**	*Mean*	*0.96*	*1.24*	*1.33*	*1.24*	*1.23*	*1.17*	*1.25*	*1.45*	*1.30*	*1.62*
	*SD*	*1.00*	*1.11*	*1.17*	*1.07*	*0.88*	*0.83*	*0.94*	*1.15*	*0.97*	*1.28*

Comparison of in vivo organ activity measured by PET (left side; mean ± standard deviation) and ex vivo activity measured in organs removed after PET. Ex vivo data of entire egg and cloaca were not assessed (NA) due to sampling practicability (see Supplementary material 3 for graphical presentation). Statistics representing the correlation between the latest in vivo measurement and ex vivo data: Spearman’s Rho: 0.9091; *p* = 0.00004; mean difference ratio: 0.92; lower limit of agreement: 0.64; upper limit of agreement: 1.2.

## Discussion

This study evaluated the feasibility of fertilized emu eggs as in vivo model for preclinical PET/CT imaging with routine scanners. The results indicated that emu eggs provided relevant information on the biodistribution of [^18^F]FDG, in analogy to the ostrich egg model [4].

Emu eggs presented some challenges compared to ostrich eggs, i.e., an opaque eggshell and smaller vessels (requiring expert manual skills), as well as a shorter breeding season. Overall, however, the emu egg model presented clear advantages compared with laboratory animals (see Table 2 for details).

**Table 2:** Advantages and disadvantages of the Emu egg model versus conventional animal testing and ostrich egg model

General advantages over conventional animal testing with mice, rats and chicken eggs	Advantages over ostrich eggs	Limitations over ostrich eggs
No need of dedicated small-animal imaging devices	Availability outside ostrich laying season	Reduced overall availability due to scarcity of emu husbandry farms
Considerably lower costs for equipment, premises, and personnel (if scanners used for clinical routine are available on site)	Longer incubation period, enabling a more extended period for examinations	Inferior incubation success
No need for mouse or rat housing facilities		Smaller egg volume and experimental surface
Larger organ size compared with mice or chicken embryos		More difficult vessel access (opaque eggshell)
Reduction of animal testing with mammals (3R)		Lower puncture success (smaller vessel diameter)
Simplified study planning, as the use of fertilized bird eggs is confirmed not to be animal testing due to strict avoiding of hatching according to German law.		

In general, large-size egg models avoid the need of dedicated imaging systems (as needed for example for chicken eggs) due to their suitability for clinical scanners. Also, avian eggs do not require full-scale husbandry, as the eggs contain all necessary nutrients and the housing management is limited to the control of temperature, humidity, and turning regime. Daily inspections are nonetheless obligatory to prevent infections, eggshell fractures, and incubator-related environmental changes [18]. In regulatory terms, avian egg models require only a fraction of the efforts required for the approval of animal experiments, provided that the embryo experiments are performed a few days before hatching.

Obtaining a sufficient quantity of emu eggs proved also more difficult compared to ostrich eggs, since emu farms are relatively scarce [14, 19]. Moreover, while a fertilization rate of 83.7% and a survival rate of 58.1% were in line with published data [6, 7], they were still lower than for ostrich eggs [14]. These rates should be considered when planning the study budget.

The mean egg weight (approximately 600 g) was also in line with published data [8, 20, 21]. The mean embryonal weight on DD 47 (approximately 360 g) was slightly lower than for full-term hatchlings [22], but this is explained by the further development expected to occur between DD 47 (end of current experiments) and DD 52–56 (hatching period in husbandry). The choice of DD 47 for PET/CT imaging was meant to maximize the organ size of the embryo while minimizing the risk of hatching, considering also that hatching under incubator conditions occurs slightly earlier (around DD 50–52) than under husbandry conditions (DD 52–56) [5, 7, 20, 21]. On DD 47, the CAM was expected to cover nearly the entire plane beneath the eggshell (Supplementary material 2) and to allow proper administration of the radiotracer [23].

Compared to the ostrich egg model, the emu eggs provide a longer time span for investigations due to nearly 10 days longer incubation, so that disease models and therapeutic responses may be observed for longer periods of time. Indeed, published results with chicken eggs indicate that the emu egg model has a considerable potential to provide valuable information in infection studies, tumor models, and drug testing [24–26].

The time-dependent distribution pattern of [^18^F]FDG in emu embryos proved similar to that of chicken embryos [27], ostrich embryos [28] and humans [29]. As expected, the highest uptake was found in liver (decreasing over time) and brain (increasing in the first 15 min). In contrast, the activity of the cloaca increased steadily due to urine production [30]. Considering the dynamics of the distribution profile, the metabolism of [^18^F]FDG in emu embryos appears to be broadly comparable to that of humans [31]. Interestingly, the extra-embryonic yolk sac (characteristic of avian eggs) was also visualized by a distinctly lower and steady [^18^F]FDG uptake. This likely reflects the primary function of this tissue: the continuous supply of nutrients to the developing embryo, paired with the phagocytic absorption of the yolk by the non-embryonic yolk sac membrane [32].

In terms of vessel identification, MRI and CT proved equivalent and can then be used interchangeably depending on their availability on site. Nonetheless, although lengthier, the MRI was superior in readily distinguishing viable from nonviable eggs (see Fig. 4B).

Compared to widely distributed CAM model, this emu study focused on the embryo itself. Experiments involving CAM are usually planned using chicken eggs, but concepts with emu and ostrich habe been published recently [3, 33]. Vice versa, embryo studies are available for chicken eggs; however, this requires dedicated small animal imaging devices, as stated above [34]. The CAM model is suitable for xenografts (tumor growth) and experiments investigating angiogenesis. The embryo model provides a whole-body model which is suitable for in-vivo biodistibution studies, i.e. in radiopharmaceutical development. A detailed comparison of both models is provided in Table 3.

**Table 3:** Comparison between common CAM model and Emu model

Feature	Emu egg model	Chicken CAM model
Egg size	Very large (~ 600 g)	Small (~ 50 g)
Imaging compatibility	Compatible with clinical scanners (PET/CT, MRI)	Requires specialized small-animal imaging equipment
Embryo development time	~ 47–52 days	~ 21 days
Vessel access	Technically challenging due to opaque shell and deeper vessels	easy access to CAM vessels through shell window
Shell transparency	Dark green, opaque shell; limits diaphanoscopy	Translucent shell; enables candling and vessel visualization
Ethical classification	Not considered animal testing if terminated before hatching (Germany/EU)	Widely accepted as non-animal testing before day 17; missing nociception before day 13 [35]
Use cases	Preclinical imaging, radiopharmaceutical testing, metabolic studies	Tumor xenografts, angiogenesis, metastasis, drug screening
Immune status	Naturally immunodeficient during first half of development	Same; allows xenografting of human tissues without rejection
Seasonal availability	Limited (autumn – spring), can complement ostrich eggs	Year-round availability
Sueccess rat	Lower due to technical complexity (e.g., catheterization)	High success rate for tumor grafting and imaging
Cost and logistics	Higher cost, fewer suppliers, more demanding incubation	Lower cost, widely available, easy to handle

## Limitations

The availability of emu eggs varies seasonally and regionally, depending on the number of local breeding farms, the respective breeding numbers and environmental influences such as weather, nutrition and quarantine of the parent birds [36]. Thus, the utility of the emu model is reliant on careful planning and coordination with breeding farms.

Intravenous catheterization in emu eggs was challenging, primarily due to the opaque eggshell and lack of visual or tactile control for the procedure. The main difficulty was to avoid perforation of the outer shell membrane during eggshell removal [4]. In addition, the emu CAM vessels are smaller and seemed more vulnerable than ostrich egg vessels. Thus, the successful completion of PET/CT studies in only 5 of 18 viable embryos is not entirely surprising, considering the attempts needed to optimize the technical approach. Three cases of 18 failed already before puncturing the vessels, as no vessel was found in the pre-marked area. In the remaining cases (10/18), complete or partial extravasation occurred due to vessel damage or catheter slipping out of the vascular lumen. Possible causes are intrinsic factors as embryonal inflammations, insufficient vascular development, deficient vascular resistance or pronounced embryonal motion [37], suboptimal proportions of needle and CAM-vessel, as well as still suboptimal candling procedures. Alternative methods for vessel visualization e.g., shell fenestration using belt sander and sandpaper [9], laser speckle contrast imaging [38], and CT imaging after narcotization similar to chicken eggs [39] should be evaluated in further examinations. Also, ultrasound imaging could be applied after removing eggshell membrane. Moreover, intravenous catheterization could be optimized by removing the eggshell on the air cell side and evaluating local vessels, by using glass capillary needles as known from CAM model experiments [40], or by testing another common methods of CAM model examinations, like induction of an artificial air cell with direct access to the CAM [41].

In the present study, a good consistency between in vivo and ex vivo data was deemed essential to estimate the validity of the emu model. However, it must be noted that embryo death by pentobarbital overdose did not coincide with complete metabolic shutdown, hence full consistency was not expected. To minimize the effects of this time lag, the embryos were sacrificed immediately after the end of PET data acquisition. While residual metabolism can be expected to continue for 10–15 min, specifically designed studies will be necessary to assess the biodistribution dynamics in this particular phase. In spite of these limitations, the strong correlation between in vivo and decay-corrected ex vivo results supports the validity of the imaging approach in emu eggs.

## Conclusions

Based on the in vivo and ex vivo biodistribution of [^18^F]FDG, the in ovo emu model seems to be a relevant option for preclinical in vivo imaging research. The use of this model in the development of new radiopharmaceuticals (alone or in seasonal alternation with the analogous ostrich egg model) may enable a significant reduction of animal experiments and experimental costs.

## Supplementary Information

Supplementary Material 1

Supplementary Material 2

Supplementary Material 3

Supplementary Material 4

Supplementary Material 5

Supplementary Material 6

## Data Availability

The datasets generated during and analysed during the current study are available from the corresponding author on reasonable request.
