# Supplementary material for: In ovo model with emu eggs as novel alternative to animal testing in preclinical imaging research
Source: EJNMMI Res. 2025 Sep 17;15:118. doi: 10.1186/s13550-025-01314-7 (PMC12443669; doi:10.1186/s13550-025-01314-7)
Supplement: Supplementary file 4 — Supplementary Material 4 [file 13550_2025_1314_MOESM4_ESM.docx]

Supplementary material 1. Incubation conditions

After weighing (Kern FCB, Kern & Sohn GmbH, Balingen-Frommern, Germany), the eggs were either immediately placed in a multistage egg incubator (Sofie 3; J. Hemel Brutgeräte GmbH, Verl, Germany) or stored at room temperature for maximum 7 days for successive incubation start. Incubation and storage were carried out horizontally. Breeding proceeded at standard conditions, i.e., constant temperature 36.4°C, relative humidity 40%, and tilting by 90° every 8 h between -45° and +45° over the horizontal plane. Stored eggs were turned over 180° along the longitudinal axis once a day to avoid sticking of embryonal structures
