# Supplementary material for: In ovo model with emu eggs as novel alternative to animal testing in preclinical imaging research
Source: EJNMMI Res. 2025 Sep 17;15:118. doi: 10.1186/s13550-025-01314-7 (PMC12443669; doi:10.1186/s13550-025-01314-7)

Supplementary material 2. Embryonal egg components adapted from French *(25)* (Created in https://BioRender.com)


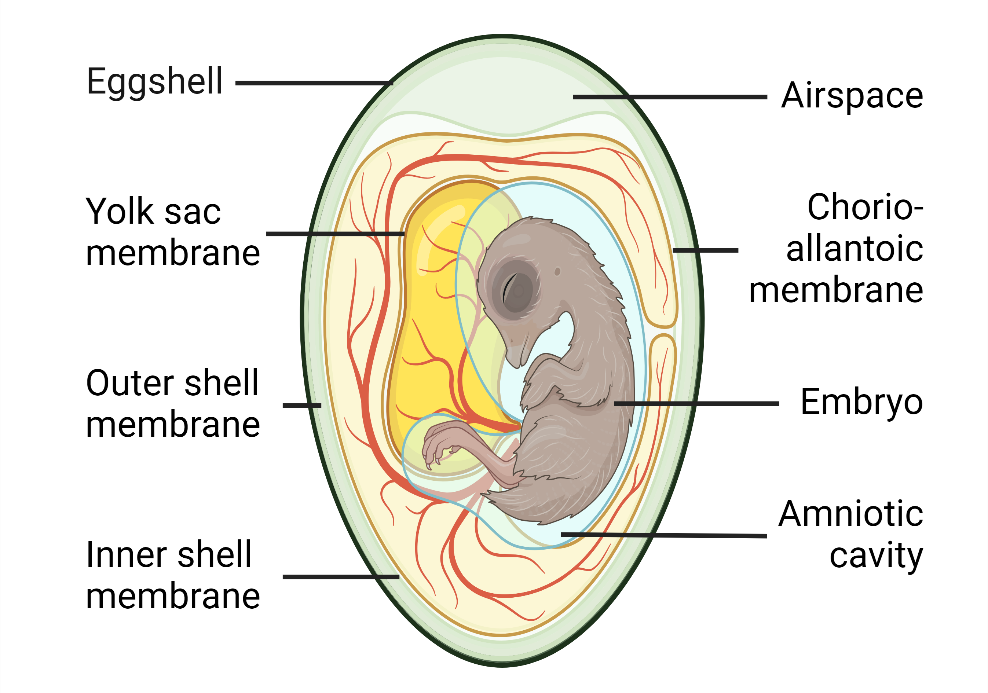

Supplement: Supplementary file 5 — Supplementary Material 5 [file 13550_2025_1314_MOESM5_ESM.docx]
