# Supplementary material for: In ovo model with emu eggs as novel alternative to animal testing in preclinical imaging research
Source: EJNMMI Res. 2025 Sep 17;15:118. doi: 10.1186/s13550-025-01314-7 (PMC12443669; doi:10.1186/s13550-025-01314-7)

Supplementary material 3. Organ activity concentration in different tissues measured in vivo by PET after administation of 10 MBq [^18^F]FDG


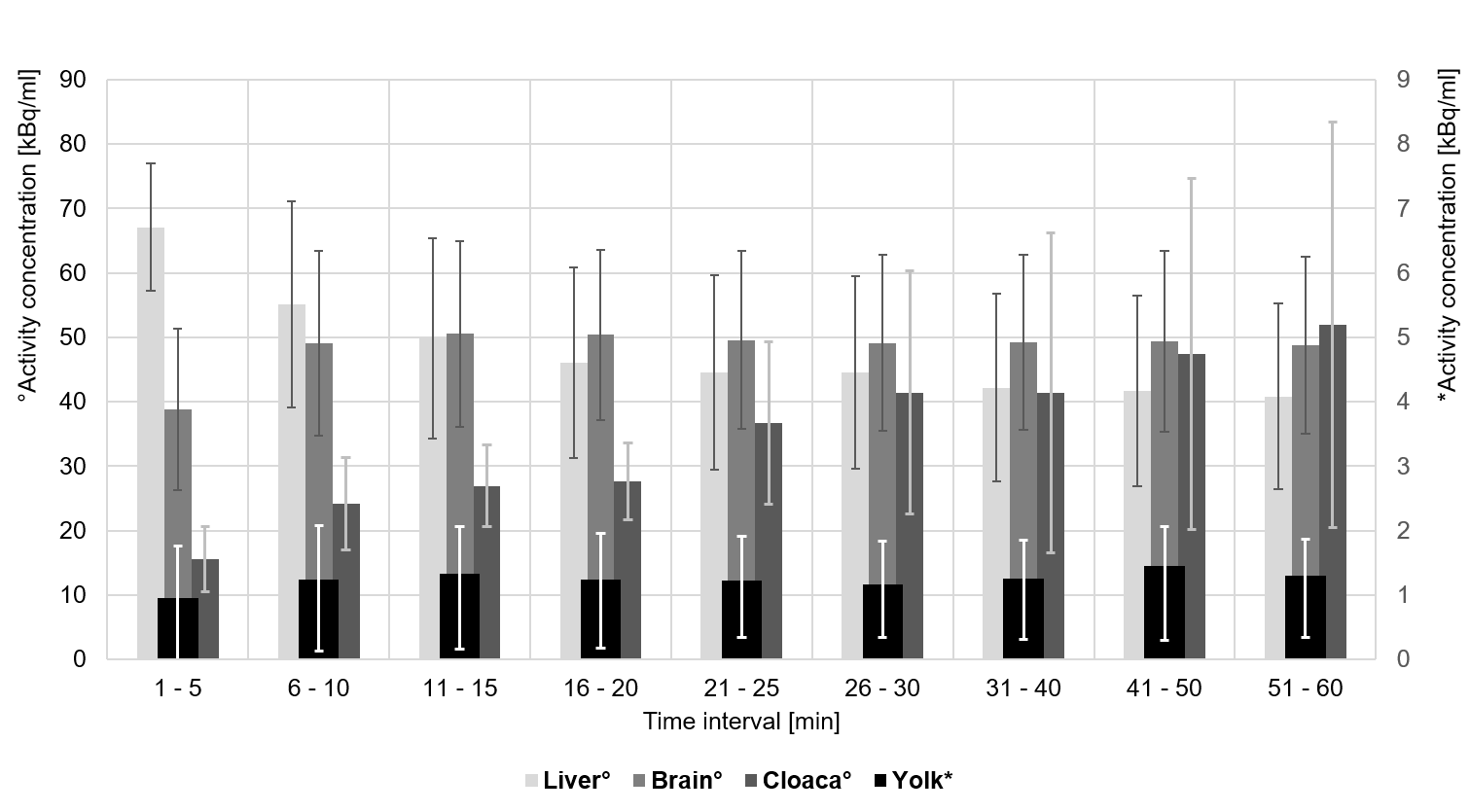

Supplement: Supplementary file 6 — Supplementary Material 6 [file 13550_2025_1314_MOESM6_ESM.docx]
